# Isolation and Characterization of Cold-Tolerant Hyper-ACC-Degrading Bacteria from the Rhizosphere, Endosphere, and Phyllosphere of Antarctic Vascular Plants

**DOI:** 10.3390/microorganisms8111788

**Published:** 2020-11-14

**Authors:** Macarena A. Araya, Tamara Valenzuela, Nitza G. Inostroza, Fumito Maruyama, Milko A. Jorquera, Jacquelinne J. Acuña

**Affiliations:** 1 Laboratorio de Ecología Microbiana Aplicada (EMALAB), Departamento de Ciencias Químicas y Recursos Naturales, Universidad de La Frontera, Ave. Francisco Salazar, 01145 Temuco, Chile; m.araya04@ufromail.cl (M.A.A.); t.valenzuela03@ufromail.cl (T.V.); nitza.inostroza@ufrontera.cl (N.G.I.); fumito@hiroshima-u.ac.jp (F.M.); milko.jorquera@ufrontera.cl (M.A.J.); 2 The Network for Extreme Environment Research (NEXER), Scientific and Technological Bioresource Nucleus (BIOREN), Universidad de La Frontera, Ave. Francisco Salazar, 01145 Temuco, Chile; 3 Center of Plant- Soil Interaction and Natural Resources Biotechnology, Scientific and Technological Bioresource Nucleus (BIOREN), Universidad de La Frontera, Ave. Francisco Salazar, 01145 Temuco, Chile; 4 Microbial Genomics and Ecology, Office of Industry-Academia-Government and Community Collaboration, Hiroshima University, Hiroshima 739-8529, Japan; 5 Center for Holobiome and Built Environment (CHOBE), Hiroshima University, Hiroshima 739-8529, Japan

**Keywords:** Antarctic vascular plants, ACC-degrading bacteria, *Colobanthus quitensis*, *Deschampsia Antarctica*, plant microbiome, plant growth-promoting bacteria

## Abstract

1-Aminociclopropane-1-carboxylate (ACC)-degrading bacteria having been widely studied for their use in alleviating abiotic stresses in plants. In the present study, we isolated and characterized ACC-degrading bacteria from the rhizosphere, phyllosphere, and endosphere of the Antarctic vascular plants *Deschampsia antarctica* and *Colobanthus quitensis*. One hundred and eighty of the 578 isolates (31%) were able to grow on minimal medium containing ACC, with 101 isolates (23, 37, and 41 endosphere-, phyllosphere- and rhizosphere-associated isolates, respectively) identified as being genetically unique by enterobacterial repetitive intergenic consensus (ERIC)-PCR. Subsequently, freeze/thaw treatments and ice-recrystallization-inhibition (IRI) activity assays were performed, the results of which revealed that 77 (13%) of cold-tolerant isolates exhibited putative ACC deaminase activity. Significant (*p* ≤ 0.05) differences in IRI activity were also observed between the studied plant niches. Surprisingly, all the cold-tolerant isolates showed ACC deaminase activity, independent of the plant niches, with 12 isolates showing the highest ACC deaminase activities of 13.21–39.56 mmol α KB mg protein^−1^ h^−1^. These isolates were categorized as ‘cold-tolerant hyper-ACC-degrading bacteria’, and identified as members of *Pseudomonas*, *Serratia,* and *Staphylococcus* genera. The results revealed the occurrence of cold-tolerant hyper-ACC-degrading bacteria in diverse plant niches of Antarctic vascular plants, that could be investigated as novel microbial inoculants to alleviate abiotic stresses in plants.

## 1. Introduction

In recent decades, climate change has become a major concern of modern agriculture, affecting crop-growing regions worldwide [1]. Importantly, plants and their interactions with associated microbes can be impacted by abiotic stresses resulting from diverse climatic adverse events, such as heat waves, droughts, flooding, and frost, negatively influencing the physiology, growth, and yields of sensitive plants [2,3,4,5]. In this context, plant–microbe interactions are known to be essential for the growth and fitness of plants, and 1-aminocyclopropane-1-carboxylate deaminase (ACC)-degrading bacteria have been widely studied in agroecosystems and proposed for use as inoculants to mitigate the adverse effects of biotic and abiotic stresses on plants in agriculture [6]. Under stressful conditions, plants increase 1-aminocyclopropane-1-carboxylate (ACC) synthesis, increasing ethylene concentrations to inhibit plant development. Interestingly, bacteria able to enzymatically degrade ACC have been shown to promote plant growth under abiotic stresses (such as drought) by lowering ethylene levels through ACC deamination [7,8,9]. Therefore, ACC-degrading bacteria have the potential to modulate the ethylene production in plants, which leads to an increase in root elongation and plant biomass production.

In recent years, the association of ACC-degrading bacteria in natural vegetation from extreme environments (e.g., hot and cold deserts) that have coevolved with the plant host and can successfully proliferate under harsh conditions have been investigated. For instance, inoculation assays with extremophilic ACC-degrading bacteria have been shown to promote the growth of plants under various stresses, such as water-deficient and high-salinity conditions [10,11]. However, most extremophilic ACC-degrading bacteria have been isolated from the rhizosphere (the portion of soil influenced by plant roots) [11], while those associated with other plant compartments, such as the endosphere (inner tissues of plants) and phyllosphere (surface of leaves) have been less studied. Similarly, salinity is currently the most-studied stress in plants inoculated with extremophilic ACC-degrading bacteria [12,13,14], while few studies have assessed the ability of these bacteria to mitigate other relevant stresses (such as cold waves and frost events), despite their importance in agriculture and the associated economic losses and social impacts [15].

The Antarctic continent is considered to be an attractive source of novel bacteria for biotechnological use in diverse fields, such as in the production of antimicrobial and antitumor compounds for biomedicine and the production of ice-binding proteins for the food industry [16,17,18]. In this context, cold-tolerant bacteria have also been shown to contribute to the survival and adaptation of plants at low temperatures, and their potential use as inoculants in agriculture to promote plant growth has also been suggested [19,20]. Recently, bacteria isolated from the phyllosphere of the Antarctic vascular plant (*Deschampsia antarctica*) and selected based on their ice-recrystallization-inhibition (IRI) activity were shown to harbor diverse genetic traits associated with plant growth promotion, such as genes involved in nutrient uptake (NH₄⁺ assimilation and N_2_ fixation) and the generation of bioactive metabolites (indole acetic acid and ACC deaminase) and antimicrobial compounds (hydrogen cyanide and pyoverdine) [21,22]. Similarly, the use of halotolerant bacteria and fungi associated with the rhizosphere and endosphere of Antarctic vascular plants (*D. antarctica* and *Colobanthus quitensis*), respectively, has also been proposed as alternative treatments to improve crop productivity in saline soils [23,24]. However, the compartmentalization of bacterial communities in plant niches (rhizosphere, endosphere, and phyllosphere) of Antarctic vascular plants was recently revealed by high-throughput DNA sequencing and showed compartment-specific differences in microbial connectivity networks and functions. In this regard, the niche differentiation of ACC-degrading bacterial populations has not been sufficiently assessed in plants living in extreme environments, particularly in Antarctic vascular plants, which may be relevant for the bioprospecting and use of cold-tolerant ACC-degrading bacteria to mitigate the impact and losses caused by cold waves and frost events in agriculture.

In the present study, culturable bacteria were isolated from the rhizosphere, endosphere, and phyllosphere of *D. antarctica* and *C. quitensis* and characterized based on ACC deaminase activity, cold tolerance, and partial 16S rRNA gene sequencing.

## 2. Materials and Methods

### 2.1. Sampling

**Figure 1:**
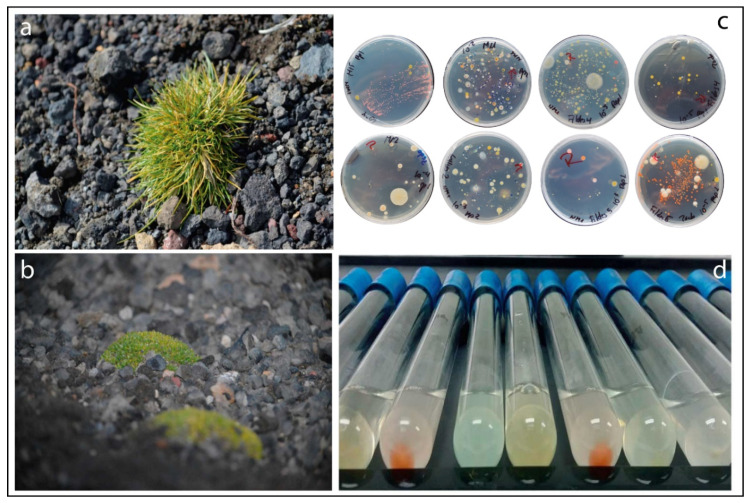
Specimens of (**a**) *Deschampsia antarctica* and (**b**) *Colobanthus quitensis* grown in Antarctic lands. (**c**) Example of phenotypic bacterial diversity isolated from plant niches. (**d**) Growth of putative 1-aminocyclopropane-1-carboxylate (ACC)-degrading bacteria in minimal medium supplemented with ACC as the sole source of nitrogen.

Sampling was performed as previously described by [25]. Briefly, specimens of *D. antarctica* and *C. quitensis* (Figure 1a,b) and their respective rhizosphere soils were collected from mantles located at the South Shetland Islands (62°59′53″ S, 60°35′17″ W and 62°24′7″ S, 58°18′29″ W, respectively) during Antarctic Scientific Expedition no. 53 (ECA53; February 2017) organized by the Chilean Antarctic Institute (INACH). The samples were placed in plastic bags, stored at 4 °C, and transported on ice to the Applied Microbial Ecology Laboratory (EMALAB) at La Frontera University.

Endosphere samples were processed in triplicate as described by Barra et al. [7], where tissues (roots and leaves) were washed and surface sterilized by repeated immersion in 70% (*v*/*v*) ethanol for 3 min and then 2.5% (*v*/*v*) sodium hypochlorite (NaOCl) treatment for 5 min, after which the samples were exhaustively rinsed with sterile distilled water. Then, tissue samples (2 g) were aseptically dissected and macerated and homogenized with a mortar and pestle before being transferred to sterile polypropylene tubes containing 10 mL of sterile saline solution (SSS; 0.85% NaCl), after which the suspensions were maintained at 4 °C. In parallel, phyllosphere leaf samples were processed in triplicate as described by Cid et al. [21], where 2-g portions of leaves were cut (aerial parts), gently washed, and vortexed for 10 min in 10 mL of SSS. Then, the leaves were removed and the recovered liquids containing detached bacterial cells were centrifuged at 7000× *g* for 5 min, suspended in 1 mL of SSS and stored at 4 °C. Similarly, rhizosphere soil samples were processed in triplicate as described by Lagos et al. [26]. Soil aggregates were detached from roots by vigorous vortexing for 10 min in 10 mL of SSS, after which the roots were then removed, and the suspensions were stored at 4 °C.

### 2.2. Isolation of Culturable Heterotrophic Bacteria

Suspensions of endosphere, phyllosphere, and rhizosphere samples stored at 4 °C were processed as described by Jorquera et al. [27]. Serial dilutions were plated onto precooled sterile NM-1 agar medium (0.5 g L^−1^ D-glucose, 0.5 g L^−1^ polypeptone, 0.5 g L^−1^ N glutamate, 0.5 g L^−1^ yeast extract, 0.44 g L^−1^ KH_2_PO_4_, 0.1 g L^−1^ (NH_4_)_2_SO_4_, 0.1 g L^−1^ MgSO_4_ × 7H_2_O, 15 g L^−1^ agar, and 1 mL of vitamin solution [1 g L^−1^ nicotinamide, 1 g L^−1^ tiamine hydrochloride, 0.05 g L^−1^ biotin, 0.5 g L^−1^ 4-aminobenzoic acid, 0.01 g L^−1^ vitamin B12, 0.5 g L^−1^ D-pantothenic acid hemicalcium salt, 0.5 g L^−1^ pyridoxamine dihydrochloride, and 0.5 g L^−1^ folic acid]), and diluted 1/10 in Luria-Bertani (LB) agar medium (1 g L^−1^ tryptone, 0.5 g L^−1^ yeast extract, 0.5 g L^−1^ NaCl, and 15 g L^−1^ agar). All agar-solidified media were supplemented with 100 μg mL^−1^ cycloheximide (Sigma-Aldrich, St. Louis, MO, USA) to prevent fungal growth. The number of total heterotrophic culturable bacteria was estimated as colony-forming units (cfu) on agar plates at each dilution after incubating for 4 to 14 days at 4 °C. Based on the colony morphotypes of colony forming units (size, edge, color, texture, and elevation) on agar plates (Figure 1c), approximately 578 representative isolates were randomly selected, purified by streaking on LB agar, and used for screening of ACC-degrading bacteria as described in the following section.

### 2.3. Selection of Putative ACC-Degrading Bacteria

The presence of culturable ACC-degrading bacteria was evaluated as described by Jorquera et al. [27]. The isolates were grown at 16 °C for 24 h in sterile tubes containing 5 mL of LB broth, after which 1 mL of each bacterial culture was transferred to sterile tubes and centrifuged at 7000× *g* for 5 min, and then the pelleted cells were repeatedly washed with 2 mL of SSS. Subsequently, the bacterial cells were suspended in 200 μL of SSS and used to inoculate sterile tubes containing 5 mL of Dworkin-Foster (DF) minimal salt medium (4.0 g L^−1^ KH_2_PO_4_, 6.0 g L^−1^ Na_2_HPO_4_, 0.2 g L^−1^ MgSO_4_ × 7H_2_O, 2.0 g L^−1^ gluconic acid, 2.0 g L^−1^ citric acid, and 1 mL of trace elements (1 mg L^−1^ FeSO4 × 7H_2_O, 10 mg L^−1^ H_3_BO_3_, 11.19 mg L^−1^ MnSO_4_ × H_2_O, 124.6 mg L^−1^ ZnSO_4_ × 7H_2_O, 78.22 mg L^−1^ CuSO_4_ × 5H_2_O, 10 mg MoO_3_). The tubes were incubated at 30 °C for 48 h under shaking (180 rpm). Then, 200-μL aliquots from tubes showing bacterial growth were transferred to sterile tubes containing 5 mL of DF minimal medium supplemented with 3 mM ACC as the sole source of nitrogen and then incubated at 16 °C for 2 to 4 days with shaking (180 rpm) (Figure 1d). Bacterial growth was monitored daily, and those that grew in ACC-supplemented DF medium were considered putative ACC-degrading bacteria.

In addition to preventing the selection of clones (colonies with similar phenotypes but different genotypes), the selected putative ACC-degrading bacteria were screened via enterobacterial repetitive intergenic consensus polymerase chain reaction (ERIC-PCR) as described by Cid et al. [21]. The selected putative ACC-degrading bacteria were grown at 30 °C for 24 h in sterile tubes containing 5 mL of LB broth, and genomic DNA was extracted from the cells using a DNeasy UltraClean Microbial kit (Qiagen N.V., Germany) according to the manufacturer’s instructions. Extracts (50 ng) were used as template for PCR using the primer set ERIC motifs 1R (5′-ATG-TAA-GCT-CCT-GGG-GAT-TCA-C-30′) and 2 (5′-AAG-TAA-GTG-ACT-GGG-GTG-AGC-G-3′). The PCR conditions were as follows: hot start at 94 °C for 5 min followed by 40 cycles of denaturation at 94 °C for 1 min, annealing at 25 °C for 1 min, and extension at 72 °C for 2 min, with a final extension step at 72 °C for 7 min. The PCR products were then run on a 2% agarose gel at 100 V for 1 h and stained with GelRed (Biotium, Fremont, CA, USA). Electrophoretic gels were photographed, and the images were analyzed using Phoretix 1D Pro gel analysis software (TotalLab Ltd., Newcastle upon Tyne, UK). One hundred and one isolates that showed different banding profiles were selected and considered to be genetically-unique putative ACC-degrading bacteria.

### 2.4. Screening of Putative ACC-Degrading Bacteria for Cold Tolerance

To assess the cold tolerance of the selected putative ACC-degrading bacteria, fresh cultures (in triplicate) were grown for 48 h in 5 mL of LB broth. Subsequently, 0.5-mL aliquots of the cultures were frozen at −20 °C for 24 h and then thawed at room temperature (18 °C). Then, 20 μL of each freeze/thaw-treated culture was spread on LB agar plates and incubated at 4 °C for 48 h. After the incubation, isolates showing growth on agar plates were preliminarily considered cold-tolerant strains and screened for ice-recrystallization-inhibition (IRI) activity.

IRI activity was also assessed as an indicator of cold tolerance for selected putative ACC-degrading bacteria using a spectrophotometry-based method described by Cid et al. [21]. Briefly, putative ACC-degrading bacteria were grown in 1/10 diluted LB broth at 16 °C for 5 days and then cold acclimatized at 4 °C for 7 days. Subsequently, the bacterial cells were harvested, and soluble cell proteins were extracted using B-PER bacterial protein extraction reagent (Thermo Fisher Scientific, Waltham, MA, USA). The isolates were screened for IRI activity at 500 nm using protein extracts (0.5–1 mg mL^−1^) in 30% sucrose solution. The microtiter plate was frozen at −80 °C for 15 min and then incubated for 2 days at −6 °C, before being assessed by spectrophotometry. Antifreeze protein (Type III AFP) was used as a positive-IRI-activity control, and protein extracts from *Escherichia coli* JM109 were used as a negative control. Microtiter plate wells with 30% sucrose solutions were used as blanks.

### 2.5. Quantification of ACC Deaminase Activity

Based on the IRI activity assay results, the ACC deaminase activity of 77 putative ACC-degrading bacteria was confirmed as described by Penrose and Glick [28], where the amount of α-ketobutyrate (α-KB) generated by the deamination of ACC was measured. The putative ACC-degrading bacteria were grown in DF medium and incubated at 16 °C for 48 h. Then, the bacterial cells were pelleted by centrifugation (10,000× *g* for 10 min), repeatedly washed with 1 mL of SSS, suspended in 600 µL of 100 mM Tris HCl (pH 8.5) and 30 μL of toluene, and then vortexed for 30 s. Then, the toluene-treated cell suspensions were incubated at 4 °C for 1 h and centrifuged at 10,000× *g*, with the crude cell extracts immediately used for total protein content analysis and enzymatic assays. The amount (μmole) of α-KB produced was determined by comparison with a standard curve prepared with known concentrations of pure α-KB measured at 540 nm (Sigma-Aldrich, St. Louis, MO, USA).

Twelve cold-tolerant isolates (>0.4 absorbance) showing the highest ACC deaminase activity values (>10 mmol α-KB mg protein^−1^ h^−1^) were designated as ‘cold-tolerant hyper-ACC-degrading bacteria’ and subjected to taxonomic analysis.

### 2.6. Taxonomic Analysis of Selected Cold-Tolerant ACC-Degrading Bacteria

The taxonomic affiliations of 12 cold-tolerant hyper-ACC-degrading bacteria were determined based on partial 16S rRNA gene sequence analysis as follows. The 16S rRNA gene fragments were amplified using the stored DNA extracts (previously used for ERIC-PCR screening) by PCR with the universal primers 27f (5′-AGA-GTT-TGA-TCC-TGG-CTC-AG-3′) and 1492r (5′-TAC-GGY-TAC-CTT-GTT-ACG-ACT-T-3′). The PCR thermocycling conditions were as follows: a hot start at 94 °C for 5 min followed 35 cycles at 94 °C for 1 min, 52 °C for 1 min, and a final extension of 72 °C for 2 min. The PCR products were sequenced using a 3500 Genetic Analyzer (Applied Biosystems^TM^; Thermo Fisher Scientific, Waltham, MA, USA) at the Scientific and Technological Bioresource Nucleus (BIOREN-UFRO), Universidad de La Frontera. The nucleotide sequences were compared with those in the GenBank database of the National Center for Biotechnology Information (NCBI) using the BLAST tool (https://blast.ncbi.nlm.nih.gov/Blast.cgi). The nucleotide sequences of the 16S rRNA gene were deposited in the GenBank database under the accession numbers MT786310 to MT786321.

### 2.7. Statistical Analysis

The data obtained in the present study were analyzed by one-way analysis of variance (ANOVA) followed by Tukey’s test using IBM SPSS Statistics version 21 (SPSS, Chicago, IL, USA). One-way ANOVA was performed to assess the differences in bacterial activities between each plant compartment. Differences were considered significant when the *p* value was less than 0.05.

## 3. Results

### 3.1. Culturable Bacterial Counts and Isolation of Putative ACC-Degrading Bacteria

**Figure 2:**
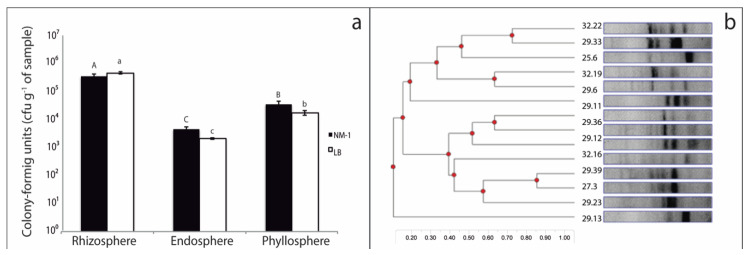
Counts of total heterotrophic bacteria (**a**) on NM-1 (light bars) and Luria-Bertani (LB) (solid bars) agar solidified medium for different plant niches. (**b**) Example of a dendrogram built using Phoretix 1D Pro gel analysis (TotalLab Ltd., Newcastle upon Tyne, UK) representing enterobacterial repetitive intergenic consensus polymerase chain reaction (ERIC-PCR) results and used to identify putative ACC-degrading bacteria with similar phenotypes but different genotypes. The bars represent standard error, and the same letter (uppercase for NM-1 or lowercase for LB) denotes no significant difference (*p* ≤ 0.05, Tukey’s multiple range test).

The cfu counts on agar plates showed significant differences (Tukey’s post-hoc test, *p* ≤ 0.05) in culturable heterotrophic bacteria among the rhizosphere, endosphere, and phyllosphere samples from both Antarctic plants (Figure 2a). The total numbers of culturable bacteria were greater in rhizosphere samples (1–10 × 10^6^ cfu g^−1^ of sample) than the phyllosphere (1–5 × 10^5^ cfu g^−1^ of sample) and endosphere (1–5 × 10^4^ cfu g^−1^ of sample) samples. It is also noteworthy that greater diversity in bacterial phenotypes was observed in the rhizosphere samples than in the endosphere and phyllosphere samples.

Based on the colony phenotype, 578 isolates were obtained from different plant niches, including 403, 89, and 86 isolates from the rhizosphere, endosphere, and phyllosphere samples, respectively (Table 1). Among these isolates, 31.1% (180) were able to grow in DF minimal medium supplemented with ACC as the sole N source, corresponding to 20.5%, 52.3%, and 58.4% of isolates from the rhizosphere, phyllosphere, and endosphere samples, respectively (Table 1). The ERIC-PCR dendrogram showed that 56% (101/180) of isolates grouped in different clusters with distinct genetic variability. One hundred and one genotypes were classified as single isolates and selected as putative ACC-degrading bacteria, including 23 isolates from the endosphere (51.1%), 37 isolates from the phyllosphere (71.15%), and 41 isolates from rhizosphere (49.4%) samples. Thus, in contrast to that observed in phenotypic diversity assessments, a higher genetic variability of putative ACC-degrading bacteria was revealed by ERIC-PCR in phyllosphere samples than in the other plant niches (Figure 2b).

### 3.2. Screening of Cold-Tolerant ACC-Degrading Bacteria and Detection of IRI Activity

**Figure 3:**
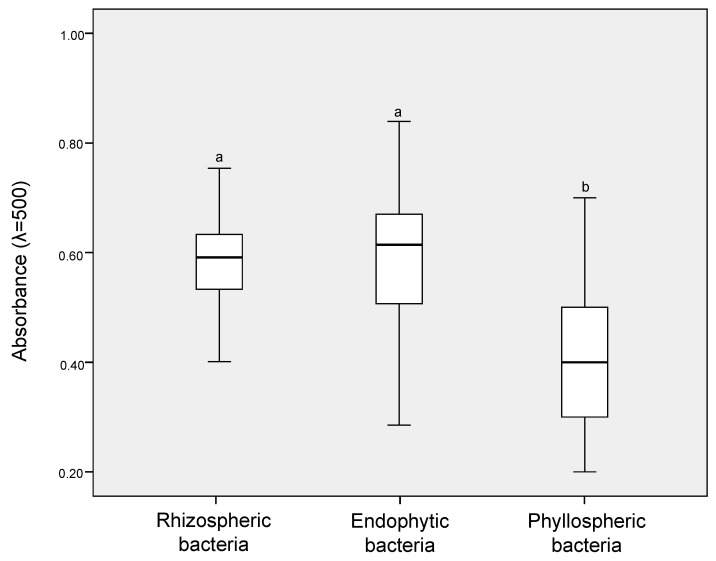
Boxplot of the putative ice-recrystallization-inhibition (IRI) activities of heterotrophic bacteria isolated from the rhizosphere, endosphere, and phyllosphere of Antarctic vascular plants. The centerline of each box represents the median, the top and bottom of boxes represent the 25th and 75th percentile of data, respectively, and the top and bottom of the error bars represent the 5th and 95th percentile of data, respectively. α-KB: α-ketobutyrate. Small letters indicate significant differences (*p* ≤ 0.05) among samples.

The cold tolerance assay results revealed that all of the selected putative ACC-degrading bacteria were able to survive freeze/thaw treatment (−20 °C for 24 h), as revealed by a high rate of growth observed on LB agar plates after cultivation at 4 °C for 48 h. In addition, in IRI activity assays, absorbance values ranging from 0.2 to 0.8 for protein extracts were obtained for the 101 isolates. Therefore, 76% (77 isolates) of putative ACC-degrading bacteria isolates were considered IRI-positive with absorbance values equal to or above 0.4 (Figure 3). However, significantly-higher IRI activities (Tukey’s post-hoc test, *p* ≤ 0.05) were observed between ACC-degrading bacteria isolated from the endosphere and rhizosphere samples compared to those isolated from the phyllosphere samples.

### 3.3. ACC Deaminase Activity of Cold-Tolerant Bacterial Isolates

**Figure 4:**
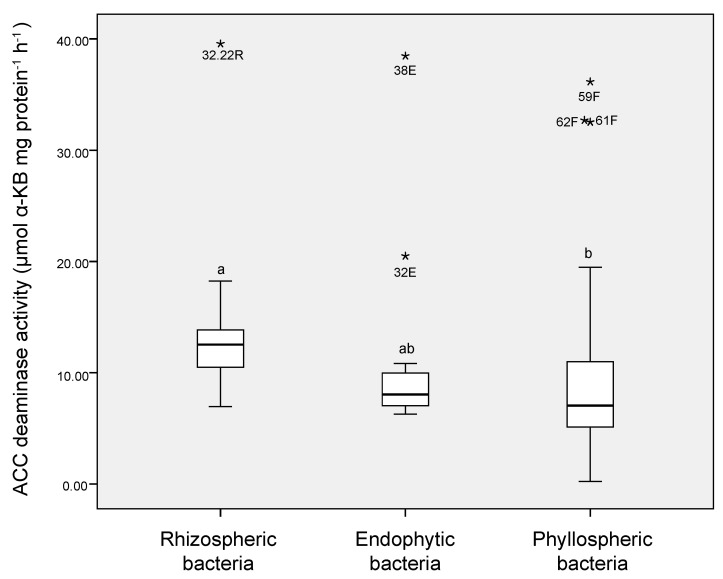
Boxplot of the 1-aminocyclopropane-1-carboxylate (ACC) deaminase activities of heterotrophic bacteria isolated from the rhizosphere, endosphere, and phyllosphere of Antarctic vascular plants. The centerline of each box represents the median, the top and bottom of boxes represent the 25th and 75th percentile of data, respectively, and the top and bottom of the error bars represent the 5th and 95th percentile of data, respectively. α-KB: α-ketobutyrate. Asterisks represent outliers. Small letters indicate significant differences (*p* ≤ 0.05) among samples.

ACC deaminase activity was assessed for 77 cold-tolerant putative ACC-degrading bacteria, with all the tested isolates showing activities that ranged from 2.26 to 39.56 mmol α KB mg protein^−1^ h^−1^ (Figure 4). Significant differences in ACC deaminase activity (Tukey’s post-hoc test, *p* ≤ 0.05) were also observed between isolates, where higher ACC deaminase activity values were observed in protein extracts from rhizobacteria (average values of 7.90 to 39.56 mmol α KB mg protein^−1^ h^−1^) and endospheric bacteria (average values of 6.29 to 38.46 mmol α KB mg protein^−1^ h^−1^) than in protein extracts from phyllospheric bacteria (average values of 2.26 to 36.15 mmol α KB mg protein^−1^ h^−1^). Notably, high ACC deaminase activity was observed in protein extracts for strain 32.22R (39.56 mmol α KB mg protein^−1^ h^−1^) and strain 38E (38.46 mmol α KB mg protein^−1^ h^−1^), which were isolated from the rhizosphere and endosphere, respectively. For the phyllosphere samples, isolates 59F, 62F, and 61F also showed the highest ACC deaminase activity in their protein extracts, with average values of 36.15, 32.53, and 32.51 mmol α KB mg protein^−1^ h^−1^, respectively (Table 2).

### 3.4. Identification of Cold-Tolerant Hyper-ACC-Degrading Bacteria

Based on our results, 12 isolates were categorized as cold-tolerant hyper-ACC-degrading bacteria and identified based on 16S rRNA sequence analysis. The taxonomic characterization of the selected endophytic bacteria, strains 38E and 32E, revealed their high similarity (>99%) with members of the *Pseudomonas* and *Ewingella* genera from cold environments, respectively (Table 2). The phyllospheric strains 59F, 62F, 61F, and M15-3A showed high similarity (>98%) with members of the genera *Pseudomonas*, *Serratia,* and *Rhanella*, respectively, which were primarily associated with plants (Table 2). Finally, the rhizospheric isolates 32.22R, 32.17R, 102R, 9.9R, 29.13R, and 2124R were taxonomically associated with members belonging to the *Serratia*, *Pseudomonas*, *Staphylococcus,* and *Enterobacter* genera, respectively, which are primarily associated with soil and cold environments (Table 2).

## 4. Discussion

Because bacterial communities associated with native plants grown in Antarctic ecosystems have coevolved with their plant hosts and local conditions, they may exhibit a wide variety of metabolic features that contribute to the adaptation of plants to nutrient-poor soils and harsh conditions. In this regard, the results of a number of studies have revealed a compartmentalization of the structure and functionality of bacterial communities in the different niches of *D. antarctica* and *C. quitensis*, where the occurrence of plant growth-promoting (PGP) bacteria can occur, influencing the tolerance of plants to abiotic stresses [25,29,30]. Studies have also demonstrated that plants under stress conditions produce and concentrate ACC in their tissues [31,32], which may result in an increased density of active ACC-degrading bacteria with a simultaneous decrease in the ACC concentration in the plant tissues [33]. In this context, Gallardo-Cerda et al. [24] isolated ACC-degrading rhizobacteria from *D. antarctica* and *C. quitensis,* and their inoculation increased the resistance of Antarctic vascular plants to salt stress. Thus, given the importance of ACC-degrading rhizobacteria to plant stress tolerance [34,35,36], additional studies are needed to understand the role of ACC-degrading bacteria in native plants living in extreme and/or cold environments. Furthermore, it is important to determine their bioprospecting potential to promote their use in improving the abiotic stresses tolerance of agricultural plants threatened by climate change-related events.

In the present study, we evaluated the occurrence of cold-tolerant ACC-degrading bacteria in diverse plant niches (rhizosphere, endosphere, and phyllosphere) of *D. antarctica* and *C. quitensis*. Significantly-higher loads of total culturable heterotrophic bacteria were observed in the rhizosphere (10^6^ cfu g^−1^ of sample) than in the phyllosphere (10^5^ cfu g^−1^ of sample) or endosphere (10^4^ cfu g^−1^ of sample) of the assayed plants. The rhizosphere has widely been reported to be a major hotspot for microbial colonization and activity in soils, harboring a huge abundance and diversity of bacteria compared with other plant and soil niches [37]. Several studies have also reported that the rhizosphere associated with Antarctic vascular plants may contain 10^6^–10^8^ bacterial cells g^−1^ [38,39,40]. In another study, Cid et al. [21] reported higher bacterial loads (10^6^ cfu g^−1^ of sample) in phyllosphere samples from *D. antarctica* than those observed in our present study. This difference could be attributed by differences in abiotic factors (ultraviolet radiation, temperature, desiccation, etc.) in the rhizosphere that can vary within a few minutes, hours, days, or even seasons [41,42]. In contrast to the phyllosphere, the endosphere is considered to be a more stable but restricted niche in plants in which colonization by bacteria depends on the degree of symbiosis between the colonizing bacteria (e.g., opportunistic or facultative) and the host plant such that only adapted populations can survive and/or proliferate in the endospheres and phyllospheres of Antarctic vascular plants. Our results showed similar bacterial loads (10^4^ cfu g^−1^ of sample) in the endosphere samples compared with those detected in two native-alpine plant species (*Oxyria digyna* and *Juncus trifidus*) from Arctic environments [43].

Bacteria can colonize and modulate plant metabolism at low and subzero temperatures, enhancing plant acclimation due to their cold-adapted mechanisms [23]. Cold-tolerant mechanisms in bacteria, such as antifreeze proteins (AFPs), including those exhibiting ice-recrystallization-inhibition (IRI) activity [44], have been described as an adaptative response of bacteria to proliferate and survive under freezing temperatures [18]. In this regard, AFP-producing strains belonging to the *Pseudomonas* genera have been shown to promote the growth and stress tolerance of Antarctic mosses at freezing temperatures [45,46]. Thus, cold-adapted PGP bacteria could contribute to ameliorating the detrimental effects of cold and freezing stress on plant performance.

Seventy-seven bacterial strains were isolated and selected as putative ACC-degrading bacteria exhibiting cold tolerance based on their ability to grow at 4 °C, survive freezing/thawing treatment (−20 °C for 48 h) and IRI activity. Psychrotolerant and psychrophilic bacteria associated with the rhizosphere of Antarctic vascular plants harboring PGP traits, such as ACC deaminase activity, have previously been reported [47,48]. Microbial adaptation to permanently-cold environments is widely known to include the optimization of basic cellular processes that are necessary for growth and survival [49]. In this context, Cid et al. [21] reported the occurrence of several PGP traits, including ACC deaminase-encoding genes in the genomes of *Pseudomonas* sp. strains isolated from the phyllosphere of *D. antarctica.* Similarly, Nascimiento et al. [50] reported the occurrence of ACC deaminase activity and the presence of the encoding gene *acdS* in the chromosome of the psychrophile marine actinobacterium *Agreia* sp. PHSC20C1 and other soil Actinobacteria isolated from Antarctic samples.

In our present study, differences in ACC deaminase activity were also observed between isolates from different plant compartments (ranging from 2.26 to 39.56 mmol α KB mg protein^−1^ h^−1^), with higher activity observed in isolates from the rhizosphere compared with the other studied niches, independent of plant species. However, to the best of our knowledge, studies comparing and reporting differences in ACC deaminase activity between bacterial isolates from different plant niches, including Antarctic vascular plants, have not been published to date.

Interestingly, in our present study, we also observed the occurrence of hyper-ACC-degrading bacteria (*Pseudomonas* sp. 38E, *Pseudomonas* sp. 59F, *Pseudomonas* sp. 62F, *Serratia* sp. 61F, and *Serratia* sp. 32.22R), with ACC deaminase values ranging from 32.5 to 39.6 nmol α KB mg protein^−1^ h^−1^. Nascimiento et al. [51] reported the screening of ACC-degrading bacteria associated with soils and different niches from plants grown in various environments (Brazil, Portugal, and Antarctica), where the strain *Pseudomonas thivervalensis* SC5 isolated from the endosphere of a *Solanum capsicoides* fruit grown in Brazil showed the highest ACC deaminase activity (18.592 μmol α KB mg protein^−1^ h^−1^). Similarly, the psychrotolerant bacterium *Pseudomonas vancouverensis* OB155 isolated from winter agricultural field soil samples was characterized as having a high ACC deaminase activity of 32.40 nmol α KB mg protein^−1^ h^−1^, and its inoculation increased chilling stress alleviation in tomato plants [52]. However, lower ACC deaminase activity is generally observed in plant-associated bacteria, including agronomic and extremophilic plants, with average values of 0.5 to 4.5 μmol α KB mg protein^−1^ h^−1^ [7,11,51].

Regarding the taxonomic affiliation of cold-tolerant hyper-ACC degrading bacteria, 5 of 12 isolates were affiliated with the *Pseudomonas* genus, members of which have been described as a common inhabitants of Antarctic plant roots [38,40,53]. A recent metagenomic study revealed that Pseudomonadaceae was the most abundant family in the endosphere and phyllosphere samples of *D. antarctica* and *C. quitensis* [25]. Similarly, Cid et al. [18] observed *Pseudomonas* as the predominant genus in the phyllosphere of *D. antarctica*. In addition, the common presence of *Pseudomonas* strains in various niches of Antarctic vascular plants has been attributed to their diverse metabolism and wide functional activity, including the degradation of organic compounds, cold tolerance, and nutrient cycling [54]. Our results also revealed the presence of the enterobacteria *Ewingella* sp. (one isolate), *Serratia* sp. (two isolates), and *Rahnella* sp. (one isolate), the closest relative of which are isolates from cold environments, according to the GenBank database (Table 2). Members of the Enterobacteriaceae family (e.g., *Enterobacter* spp., *Serratia* and *Rahnella*) have been frequently isolated from Antarctic and Arctic environments, including Antarctic plants and soils [24,47,48,55,56]. It is noteworthy that *Ewingella* sp. has not been previously reported to have been isolated from either plants or soils from Antarctica. *Staphylococcus* spp. from the *D. antarctica* rhizosphere was also detected. da Silva et al. [57] reported the isolation of *Staphylococcus* sp. strain LOCK 1005 from the *C. quitensis* rhizosphere. Finally, significant differences in terms of strains diversity, IRI activity, and ACC-deaminase activity were observed between plant niches, independent of plant species. In this context, Zhang [25] reported significant differences of bacterial community composition between niches of Antarctic vascular plants, but these differences were not observed among plant species.

During recent years, cold-tolerant PGP bacteria have attracted attention for their potential role in plant growth at lower temperatures and as inoculants to mitigate the impact of chilling and freezing events in agriculture. Considering that low temperatures can induce an increase in ACC in tissues as a result of ethylene stress, plants may select efficient ACC-degrading bacteria to counteract the associated negative effects. Thus, the use of hyper-ACC-degrading bacteria appears to be an attractive strategy to promote the rapid adaptation of plants to changing environmental conditions. This is the first study reporting the simultaneous isolation of cold-tolerant hyper-ACC-degrading bacteria associated with different niches of Antarctic vascular plants, providing clues regarding their differences and demonstrating a potential source of new PGP bacteria to alleviate abiotic stresses in agronomic plants. However, additional studies are needed to elucidate the role of cold-tolerant hyper-ACC-degrading bacteria in Antarctic vascular plants and agronomic plants under climate change scenarios.

## 5. Conclusions

Our results demonstrated the association of 1-aminocyclopropane-1-carboxylate deaminase (ACC)-degrading bacteria with the rhizosphere, endosphere, and phyllosphere of specimens of *Deschampsia antarctica* and *Colobantus quitensis*. Among 578 isolates, 77 (13%) were found to possess both putative ACC deaminase activity and production of the ice-recrystallization-inhibition activity (IRI). The ACC-degrading strains isolated from rhizospheric, endospheric, and phyllospheric samples were characterized as members of *Pseudomonas*, *Serratia,* and *Staphylococcus* genera with an ACC deaminase activity range of 13.21 to 39.56 mmol α KB mg protein^−1^ h^−1^. The results suggest that the isolated strains denominated as ‘cold-tolerant hyper-ACC-degrading bacteria’ can be used in future studies focusing on plant stress tolerance and plant growth promotion, as well for development of a new generation of stress-adapted inoculants in order to prevent deleterious effects caused by abiotic stresses (e.g., salinity and drought) in different crops and/or cultivars.

## Figures and Tables

**Table 1:** Selection of putative ACC-degrading bacteria isolated from different niches of Antarctic vascular plants.

Niche	No. of Total Isolates	Growth in DF + ACC		ERIC-PCR Genotyping	
		No. of Isolates	%	No. of Isolates	%
Endosphere	86	45	52.32	23	51.11
Phyllosphere	89	52	58.42	37	71.15
Rhizosphere	403	83	20.59	41	49.40
Total	578	180	31.14	101	56.11

DF + ACC: Dworkin-Foster (DF) minimal medium supplemented with 1-aminocyclopropane-1-carboxylate (ACC) as sole source of nitrogen; ERIC-PCR: enterobacterial repetitive intergenic consensus polymerase chain reaction.

**Table 2:** Characterization of selected cold-tolerant hyper-ACC-degrading bacteria isolated from Antarctic vascular plants (*Deschampsia antarctica* and *Colobanthus quitensis*) by partial sequencing the 16S rRNA gene.

Niche/Isolate	Plant	Closest Relatives or Cloned Sequences (Accession No.; % of Identity) *	Accession No.	ACC-Deaminase ^Ɏ^	IRI ^§^
*Endosphere*					
38E	*D. antarctica*	*Pseudomonas* sp. PCH176 from soil of Himalayan region (MF774162; 100%)	MT786310	38.46 ^¥^ a	0.80 a
32E	*D. antarctica*	*Ewingella* sp. strain 3–24 from leaves collected from cold water pools of Huanglong park (KX378962; 99%)	MT786318	20.49 b	0.57 b
*Phyllosphere*					
59F	*D. antarctica*	*Pseudomonas* sp. L3_E02 from phyllosphere of *Carpinus betulus* L (MK216837; 100%)	MT786317	36.15 a	0.76 a
62F	*D. antarctica*	*Pseudomonas gessardii* strain P_B71/5 from Baltic sea (MT626814; 98%)	MT786316	32.53 a	0.49 b
61F	*C. quitensis*	*Serratia marcescens* strain BRM 046341 from corn stigma (MK461848; 100%)	MT786319	32.51 a	0.55 b
M15-3A	*D. antarctica*	*Rahnella* sp. strain UASWS1864 from oak in Geneva (MK513744; 99%)	MT786312	14.08 b	0.86 a
*Rhizosphere*					
32.22R	*D. antarctica*	*Serratia* sp. JSC-N623-1 from soil (JF958141; 99%)	MT786315	39.56 a	0.81 a
32.17R	*C. quitensis*	*Pseudomonas* sp. strain C14-10 from sediment (MT255246; 99%)	MT786314	18.24 b	0.59 b
10.2R	*D. antarctica*	*Staphylococcus* sp. CSA7 from soil (HQ437165.1; 99%)	MT786311	16. 34 b	0.57 b
9.9R	*D. antarctica*	*Staphylococcus condimenti* strain HBUAS56223 from vegetables (MT229638; 100%)	MT786321	16.12 b	0.59 b
29.13R	*C. quitensis*	*Enterobacter* sp. CV87 from soil (KJ482866; 99%)	MT786320	13.89 b	0.65 b
21.24R	*C. quitensis*	*Pseudomonas* sp. strain PAMC 27327 from Antarctic soil (MT555366; 99%)	MT786313	13.21 b	0.66 b

* Taxonomic affiliation based on partial sequencing of 16S rRNA gene and comparison with those present in GenBank by using BLASTn; ^¥^ Values are means ± standard error of three experiments. Small letters represent significant differences (*p* ≤ 0.05) among samples from each plant niches; ^Ɏ^ 1-aminocyclopropane-1-carboxylate (ACC) deaminase activity measured as the μmole of α-ketobutyrate mg protein^–1^ h^–1^ generated by the hydrolysis of ACC; ^§^ putative ice-recrystallization-inhibition (IRI) activity measured as absorbance at 500 nm. *E. coli* used as negative control presented an absorbance value of 0.4.
